# In Situ Profiling of the Three Dominant Phyla Within the Human Gut Using TaqMan PCR for Pre-Hospital Diagnosis of Gut Dysbiosis

**DOI:** 10.3390/ijms21061916

**Published:** 2020-03-11

**Authors:** Young Jae Jo, Setu Bazie Tagele, Huy Quang Pham, YeonGyun Jung, Jerald Conrad Ibal, SeungDae Choi, Gi-Ung Kang, Sowon Park, Yunkoo Kang, Seung Kim, Hong Koh, Jae-Ho Shin

**Affiliations:** 1 School of Applied Biosciences, College of Agriculture and Life Sciences, Kyungpook National University, Daegu 41566, Korea; dudwo7573@naver.com (Y.J.J.); setubazie@gmail.com (S.B.T.); huypham@knu.ac.kr (H.Q.P.); jyg1076@knu.ac.kr (Y.J.); jerald.ibal@gmail.com (J.C.I.); csd506@knu.ac.kr (S.C.); gukang@knu.ac.kr (G.-U.K.); 2 Pediatric Gastroenterology, Hepatology and Nutrition, Severance Pediatric IBD Research Group, Severance Children’s Hospital, Yonsei University College of Medicine, 06229 Yonsei, Korea; sowon81@yuhs.ac (S.P.); hollycow@yuhs.ac (Y.K.); pedks@yuhs.ac (S.K.); KHONG@yuhs.ac (H.K.)

**Keywords:** gut microbiota, next generation sequencing, primer design, qPCR, TaqMan probes, ulcerative colitis

## Abstract

A microbial imbalance called dysbiosis leads to inflammatory bowel disease (IBD), which can include ulcerative colitis (UC). Fecal microbiota transplantation (FMT), a novel therapy, has recently been successful in treating gut dysbiosis in UC patients. For the FMT technique to be successful, the gut microbiota of both the healthy donors and UC patients must be characterized. For decades, next-generation sequencing (NGS) has been used to analyze gut microbiota. Despite the popularity of NGS, the cost and time constraints make it difficult to use in emergency services and activities related to the periodic monitoring of microbiota profile alterations. Hence, in this study, we developed a multiplex TaqMan qPCR assay (MTq-PCR) with novel probes to simultaneously determine the relative proportions of the three dominant microbial phyla in the human gut: Bacteroidetes, Firmicutes, and Proteobacteria. The relative proportions of the three phyla in fecal samples of either healthy volunteers or UC patients were similar when assessed NGS and the MTq-PCR. Thus, our MTq-PCR assay could be a practical microbiota profiling alternative for diagnosing and monitoring gut dysbiosis in UC patients during emergency situations, and it could have a role in screening stool from potential FMT donors.

## 1. Introduction

There are more than trillions of microorganisms inhabiting the human gut, and they live in homeostatic balance [1,2]. However, a microbial imbalance called dysbiosis causes metabolic disorders and affects the interactions of microorganisms in the host, which can eventually lead to inflammatory bowel disease (IBD) and ulcerative colitis (UC) [1,3]. The increasing global incidence of UC is growing, as is the concern for treating the disease [4,5].

A novel therapy named fecal microbiota transplantation (FMT) has recently shown promise in treating gut dysbiosis in UC patients [6,7,8]. However, it is difficult to determine the safety and efficacy of FMT in UC patients, and it is also difficult to determine the factors that affect the success of FMT [9,10]. The success of FMT may be associated with microbial shift [11,12]. Previous studies have characterized gut dysbiosis as an alteration in the relative abundance of the two dominant phyla in the human gut, Bacteroidetes and Firmicutes [13]. Thus, for the FMT technique to be successful, it is crucial to characterize and monitor the gut microbiota of UC patients [6,9].

For decades, next-generation sequencing (NGS) has been used to analyze changes in gut microbiota composition, and it has played a critical role in advancing gut microbiome research [14,15,16]. Despite the popularity of NGS, the cost and time constraints make it difficult to use for emergency services. A rapid, sensitive, and cost-effective method is required. Conventional and quantitative PCR methods have been useful to specifically detect organisms at the phylum level [17]. However, these methods are not able to simultaneously quantify different phyla in a single tube. TaqMan PCR is a powerful tool for the simultaneous detection and quantification of microbes in samples from different sources [18,19,20]. The relative composition of the three major phyla in the human gut, Bacteroidetes, Firmicutes, and Proteobacteria, has been proposed as a potential diagnostic tool for UC [9,21]. This study aimed to develop an MTq-PCR assay using novel TaqMan probes for profiling the dominant gut microbiota phyla. Our study confirmed that MTq-PCR is a viable alternative to NGS when characterizing the three dominant phyla (Bacteroidetes, Firmicutes, and Proteobacteria) in fecal samples from healthy and UC patients.

## 2. Results

### 2.1. Specificity of TaqMan Probes and Primers

In this study, phylum-specific probes (Table 1) were developed, and their phylum specificity was evaluated in silico using the SILVA database (Quast et al., 2013). The Bacteroidetes TaqMan probe (Bat1) matched 93% of the 16S rRNA genes that were classified under Bacteroidetes in the SILVA database when two mismatches per probe were allowed (Table 2). The Bat1 probe had low sensitivity (3.7%) to non-target 16S rRNA gene sequences classified under Firmicutes (Table 2). Interestingly, the Bat1 probe had no predicted specificity to Enterobacteriaceae, which are the gut-dominant Proteobacteria. The previous CFB555f and 798cfbF probes covered a large percentage (96.7 and 98.2%, respectively) of Bacteroidetes target sequences; however, both showed very weak mismatching capabilities against non-targets such as Firmicutes and Proteobacteria (Table 3 and Table 6).

The Firmicutes probe (Fir7) covered 55.7% of its intended phyla but had very low coverage (less than 4%) for the non-targeted phyla (Table 2). Previously reported Firmicutes-specific probes had high (>96%) coverage for Firmicutes target sequences; however, they showed very weak mismatching capabilities against non-target gene sequences from Bacteroidetes and Proteobacteria (Table 4 and Table 6). Interestingly, the Proteobacteria probe (Pro3) was not predicted to bind to non-targeted phyla. Pro3 was highly specific (94%) to Enterobacteriaceae (Table 2). Although the Pro3 probe had lower Proteobacteria coverage than the previous Proteobacteria-specific probe, our probe had comparable efficacy in terms of gut-dominant Proteobacteria (Enterobacteriaceae). More importantly, our probe showed strong mismatching capabilities against non-targeted genes from Firmicutes and Bacteroidetes (Table 5 and Table 6). Overall, our probes had very low coverage (less than 4%) to non-targeted taxa (Table 2). The three probes were designed to bind within the 16S rRNA region of the forward (Eub268) and reverse (Eub797) primers without overlapping (Figure 6).

### 2.2. In Vitro Evaluation of the Multiplex Taqman Assay

**Figure 1:**
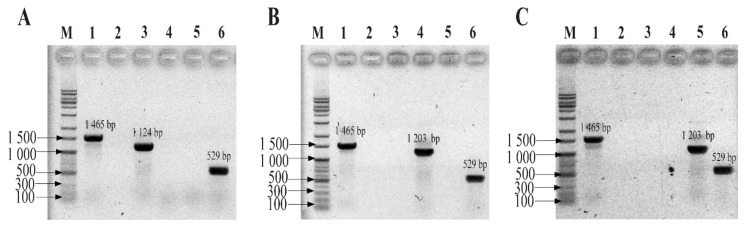
Agarose gel electrophoresis of singleplex PCR products produced by phylum-specific probes for (**A**) *Bacteroides fragilis* GUT-04, (**B**) *Clostridium butyricum* TO-A, and (**C**) *Shigella sonnei* KCCM 41282. M: marker 1kb DNA ladder; Lane 1: 27F/1492R; Lane 2: negative control; Lane 3: Bat1/1492R; Lane 4: Fir7/1492R; Lane 5: Pro3/1492R; and Lane 6: Eub268/Eub797. The universal primer set, 27F/1492R, without template DNA, served as the negative control.

**Figure 2:**
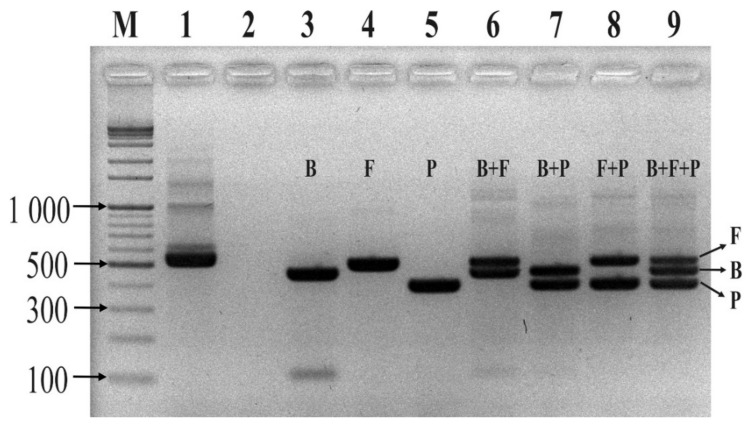
Agarose gel electrophoresis of multiplex PCR products. Letters B, F, and P signify the DNA template extracted from *B. fragilis* GUT-04, *C. butyricum* TO-A, and *S. sonnei* KCCM 41282, respectively. B+F, B+P, F+P, and B+F+P represent mock communities. M: marker 1kb DNA ladder; Lane 1: positive control; Lane 2: negative control; Lane 3: Bat1+Eub797; Lane 4: Fir7+Eub797; Lane 5: Pro3+Eub797; Lane 6: Bat1+Fir7+Eub797; Lane 7: Bat1+Pro3+Eub797; Lane 8: Fir7+ Pro3+Eub797; and Lane 9: Bat1+Fir7+Pro3+Eub797. A eubacterial primer set, Eub268/Eub797, with template DNA from the mock community without probes served as the positive control, and a primer set, 27F/1492R, without template DNA, served as the negative control.

The specificity of the probes used to detect three bacteria strains (*Bacteroides fragilis* GUT-04, *Clostridium butyricum* TO-A, and *Shigella sonnei* KCCM 41282) within different phyla was evaluated in a singleplex and multiplex conventional PCR reaction. In the singleplex PCR assay, primer pairs for Eubacteria, Eub268, and Eub797 successfully amplified a single band of 529 bp from each of the three bacterial strains (Figure 1A–C). However, the probe designed for Bacteroidetes, Bat1, only amplified the target strain, *B*. *fragilis*, and no PCR product was detected for non-target strains. Similarly, the Fir7 and Pro3 primers only amplified their intended target strain (Figure 1B and C), suggesting that the probes were specific. In the multiplex PCR, wherein a mock community of three strains was constructed, the probes specifically amplified their target strains, and the probes were sensitive enough to discriminate all strains mixed in a single tube (Figure 2). These results indicated that the designed primer and probe sets did not interfere with each other in the multiplex PCR reaction. In contrast, primers designed to detect Eubacteria failed to discriminate between the strains used in the PCR reaction (Figure 2).

### 2.3. Comparison of Multiplex TaqMan qPCR (MTq-PCR) and NGS

**Figure 3:**
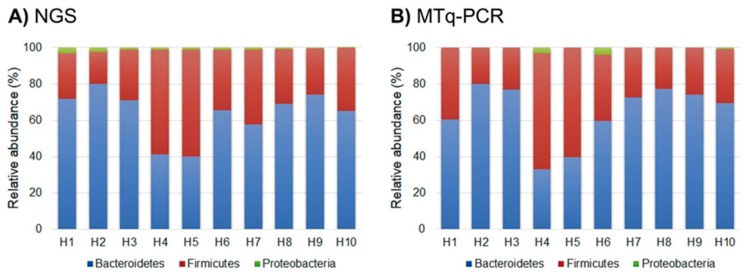
Relative proportions of the three phyla from fecal samples of healthy subjects determined by (**A**) NGS and (**B**) MTq-PCR.

**Figure 4:**
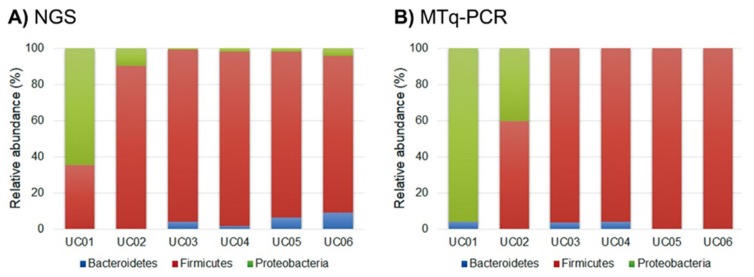
Relative proportions of the three phyla from fecal samples of UC patients determined by (**A**) NGS and (**B**) MTq-PCR.

The sensitivity of the MTq-PCR and NGS methods in determining the relative proportions of the three phyla from fecal samples collected from healthy and UC patients were compared. The relative proportions of the three phyla in fecal samples of either healthy volunteers or UC patients were similar for both methods. In fecal samples from healthy individuals, the relative proportions of Bacteroidetes and Firmicutes were much higher than the proportion of Proteobacteria. In contrast, in UC patients, both methods showed that the percentage of Bacteroidetes was very low. In both methods, the Firmicutes/Bacteroidetes ratio was close to 0.5 in fecal samples of healthy individuals, but the ratio was very high in UC patients. More importantly, both methods revealed that the proportion of Proteobacteria was high in UC fecal samples when compared to healthy individuals (Figure 3 and Figure 4).

**Figure 5:**
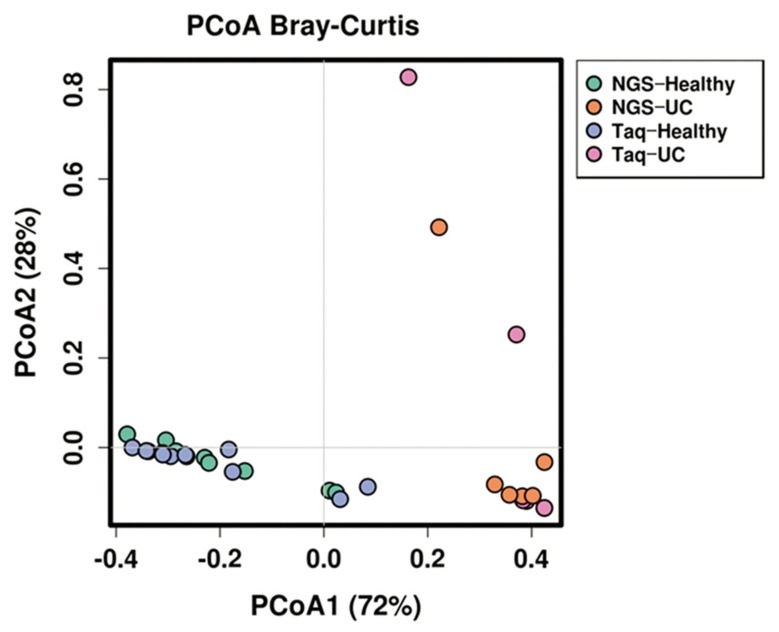
Principal coordinate analysis of (PCoA) of the three dominant phyla in the fecal samples of UC patients and healthy volunteers using NGS and MTq–PCR.

Principal coordinate analysis (PCoA) was performed to compare the profiling efficiency of NGS and MTq-PCR (Figure 5). The relative proportions of the three dominant phyla in UC patients and healthy volunteers were used for the analysis. The clustering of UC patients and healthy volunteers using NGS was similar to the clustering seen when using MTq-PCR, which indicated the potential of MTq-PCR for monitoring microbiota profile alterations.

Furthermore, the statistical analysis examining the similarity between NGS and MTq-PCR was analyzed using multivariate similarity Anosim and Adonis tests with the Bray–Curtis distance. Based on the Anosim and Adonis analysis, NGS and MTq-PCR were not significantly different (*p* > 0.05) in profiling the abundance of the three phyla from fecal samples of either healthy volunteers or UC patients (Table 7). Both NGS and MTq-PCR were able to significantly discriminate (*p* < 0.001) the phylum composition between healthy volunteers and UC patients (Table 8).

## 3. Discussion

Ulcerative colitis (UC) is a chronic inflammatory bowel disease (IBD) that adversely affects the quality of a patient’s life [27,28]. Recent reports have indicated that UC is characterized by a low diversity of intestinal bacterial flora [29,30]. The relative composition of the three major gut flora phyla (Bacteroidetes, Firmicutes, and Proteobacteria) could be a potential diagnostic tool for UC [9,21]. Fecal microbiota transplantation (FMT) is a novel therapy for UC that restores the composition and function of the bacterial flora in the gut [31,32]. For FMT to be successful, it is crucial to characterize the presence and absence of individual taxa in the samples of potential fecal donors and UC patients [33]. For decades, next-generation sequencing (NGS) has been used to analyze gut microbiota [34]. Despite the popularity of NGS, the cost and time constraints make it difficult for emergency services to employ it [33,34,35]. In addition, and of concern because the number of UC patients is rapidly increasing [36], it is more costly to monitor the clinical efficacy of FMT (i.e., analyzing the gut microbiota) of numerous people with NGS. A rapid, sensitive, and cost-effective method is required [20].

TaqMan qPCR is a powerful tool used in the microbial diagnosis of samples from different sources [19]. Hence, in this study, we developed a multiplex TaqMan qPCR (MTq-PCR) with three novel phylum-specific TaqMan probes to determine the proportions of the three dominant phyla in the gut microbiota of UC patients (*n* = 6) and healthy subjects (*n* = 6), namely Bacteroidetes, Firmicutes, and Proteobacteria. The effectiveness of our MTq-PCR assay in profiling the three phyla was compared with NGS.

The phylum-specific probes and primers were designed to target 16S rRNA and were evaluated in silico using the SILVA database [37]. Our probes were comparatively more sensitive than the previously reported probes in discriminating non-target phyla, and they were more specific to gut microbiota [24,25,26]. This indicates that our probes detected more gut microbiota than the previously reported probes. Our probes exhibited low cross-hybridization to non-target phyla. In addition, phylum-specific primers from previous reports were designed for a singleplex PCR and were less sensitive at discriminating non-target taxa [17,24]. In our conventional PCR assay, none of the designed primers and probes interfered with each other during the multiplex PCR reaction, and the reaction was confirmed to be reproducible and sensitive. The multiplex assay showed that the probes were specific, and it was possible to discriminate the three phyla in a single tube. Similar studies reported that real-time PCR assays based on TaqMan hydrolysis probes were specific, sensitive, and rapid compared to conventional PCR [20,37,38]. It is important to note that our qPCR assay was based on the proportion of amplicons; therefore, it cannot directly measure the number of individual cells. This is due to the fact that multiple 16S rRNA copies are found in different bacteria [39]. In connection with multiple copies of 16S rRNA, previous studies similarly reported a biased determination of microbial composition using an NGS approach [40]. To the best of our knowledge, this is the first study to profile human gut microbiota with three different phylum-specific TaqMan probes in a single assay without cross-reactivity.

In our study, both NGS and multiplex PCR methods showed a similar relative abundance between the three major phyla in fecal samples of either UC patients or healthy subjects. Both profiling methods also confirmed that the proportion of Bacteroidetes was greatly reduced in all fecal samples of UC patients when compared to healthy subjects. On the other hand, both methods proved that the relative abundance of Proteobacteria was very high in UC patients when compared to healthy subjects. The results of our study were consistent with a previous report showing that Proteobacteria are integral in the formation of UC, and the load of this phylum in the gut could be an effective diagnostic criterion for UC [21]. Bacteroidetes and Firmicutes are the two dominant phyla in healthy gut microbiota, representing about 90% of the bacterial population [13,40,41]. However, dysbiosis (a microbial imbalance) leads to ulcerative colitis (UC) [3,9]. In our study, both NGS and MTq-PCR showed similar results in determining dysbiosis. PCA and an Anosim and Adonis analysis revealed that NGS and MTq-PCR were not significantly different (*p* > 0.05) in profiling the abundance of the three phyla from fecal samples of either healthy volunteers or UC patients. Both NGS and MTq-PCR indicated significant differences (*p* < 0.001) in the phylum composition between healthy and UC patients. This indicates that our MTq-PCR assay could be used as an alternative method in profiling the three dominant phyla of the gut microbiota since the assay is less costly, rapid, and more accessible than NGS. NGS techniques are costly, time-consuming, and complex for routine applications in resource-limited health care organizations with limited genomic facilities and trained personnel [42].

In summary, we developed a rapid, sensitive, and cost-effective MTq-PCR assay that reliably profiled the three major phyla in fecal samples of healthy subjects and UC patients: Bacteroidetes, Firmicutes, and Proteobacteria. The results revealed that the proportion of the three phyla in either healthy subjects or UC patients were similar among NGS and MTq-PCR. This suggests that our assay could be a practical microbiota profiling alternative that can characterize the three phyla involved with gut dysbiosis in UC patients during emergency cases of pre-FMT, and it could also help monitor the clinical efficacy and safety of FMT in UC patients at a low cost.

## 4. Materials and Methods

### 4.1. Bacterial Strains

Three bacterial strains (*B. fragilis* GUT-04, *C. butyricum* TO-A, and *S. sonnei* KCCM41282) representing the three dominant gut flora phyla (Bacteroidetes, Firmicutes, and Proteobacteria, respectively) were obtained from Kyungpook National University culture collections. The strains were grown in fastidious anaerobe broth medium (FAB: peptone, 23 g; soluble starch, 1 g; sodium bicarbonate, 0.4 g; sodium chloride, 5 g; glucose, 1 g; sodium pyruvate, 1 g; L-arginine, 1 g; L-cysteine HCL 0.5 g; sodium pyrophosphate, 0.25 g; sodium succinate, 0.5 g; Hemin, 0.01 g; and Vitamin K, 0.001 g; Seoul, South Korea) at 37 °C for 48 h.

### 4.2. Fecal Sample Collection

Fresh fecal samples from healthy volunteers (*n* = 10) were collected at Kyungpook National University (KNU), Daegu, South Korea. In addition, fecal samples from six UC patients (*n* = 6) who had been diagnosed with dysbiosis at Yonsei University, Seoul, South Korea were collected. Collection of human fecal samples was performed with the approval of the Institutional Review Board (IRB) at Yonsei University and Kyungpook National University (permit numbers: YSU4-2018-0438 and KNU-2019-0129, respectively). All fecal samples were collected in Transwab^®^ (Medical Wire, UK) following the manufacturer’s protocol. Within 24 h, all collected samples were transported to our laboratory in ice packs and stored at −70 °C until processing.

### 4.3. DNA Extraction

Genomic DNA from pure cultures of the three bacterial strains was extracted using the Wizard^®^ Genomic Purification Kit (Promega Corporation, USA). DNA from fecal samples was extracted using the QIAamp Powerfecal DNA kit (QIAGEN, Germany) according to the manufacturer’s protocol. The DNA concentration was measured using a Qubit^®^ Fluorometer (Thermo Fisher Scientific, Waltham, MA, USA) and stored at −20 °C until used.

### 4.4. NGS Method

The V4–V5 variable region of the 16S rRNA gene was PCR amplified using a universal primer pair: forward (515F 5′-GTGCCAGCMGCCGCGG-3′) and reverse (907R 5′-CCGTCAATTCMTTTRAGTTT-3′). For sequencing, an Ion Torrent PGM adapter and barcode tailored to the primer pair was used. The library preparation reactions consisted of 50 μL and were composed of 25 μL of EmeraldAmp^®^ Max PCR Master Mix (Takara Korea Biomedical Inc., Seoul, Korea), 1 μL of each bacterial primer, 1 μL of extracted DNA, and 22 μL of ultra-pure water. The first amplification reaction was performed using the following thermocycling program: pre-denaturation at 95 °C for 3 min, followed by five cycles of 95 °C for 30 s, 57 °C for 30 s, and 72 °C for 30 s. The second amplification reaction was 30 cycles of denaturation at 95 °C for 30 s and annealing–extension at 72 °C for 1 min. The final extension was performed at 72 °C for 5 min [43].

In this study, NGS was employed, and the quality of the amplified DNA library was assessed using an Agilent 2100 Bioanalyzer High-Sensitivity DNA Assay kit (Agilent Technology, Santa Clara, CA, USA). The pre-amplified DNA library was further diluted to 6 pM to perform emulsion PCR with Ion Sphere™ Particles (ISPs) using the Ion OneTouch System II (Thermo Fisher Scientific Korea Inc., Seoul, Korea) followed by enrichment of template-positive ISPs with Dynabeads™ MyOne™ streptavidin C1 beads (Thermo Fisher Scientific, Waltham, MA, USA). Each sample was loaded on an Ion 316 Chip Kit v2 bar-coded chip. Sequencing was performed on the Ion Torrent PGM for 1200 flows with an Ion PGM™ Hi Q Sequencing Kit (Thermo Fisher Scientific Korea Inc., Seoul, Korea). The Torrent Suite™ and Ion Torrent PGM-specific pipeline software was employed to generate sequence reads, trim adapter sequences, filter, and remove low-quality signal-profile reads. Quality filtering of generated sequences and taxonomic classification were performed using QIIME software based on the Greengenes database [44].

### 4.5. TaqMan Probe and Primer Design

Five bacterial species from the three dominant phyla in the human gut (Bacteroidetes, Firmicutes, and Proteobacteria) were used for probe design (Table 9.). Complete 16S rRNA gene sequences from 15 strains representing the three phyla were downloaded from the National Center for Biotechnology Information (NCBI) GeneBank database. Multiple sequence alignments of the 16S rRNA gene sequences from the 15 strains were performed using CLC Main Workbench 8.1 software (Qiagen, Hilden, Germany). Following alignment, a primer pair targeting the 16S rRNA of Eubacteria was designed from a conserved region of all strains, and phylum-specific TaqMan probes were selected from the consensus sequence of each phylum. All probes were designed to be within the binding site of the eubacteria primer pair. A universal probe for eubacteria that targets the opposite strand of the conserved region of the three phyla was used [22]. The specificity of the primer pair and TaqMan probes were confirmed by in silico analysis using the SILVA database [37].

### 4.6. Conventional PCR Assay

Before conducting the multiplex TaqMan qPCR (mTqPCR) assay, conventional PCR assays were performed to determine the specificity of the designed primer/probe sets using a Mastercycler Nexus PCR machine (Eppendorf, Hamburg, Germany). The primer pairs and TaqMan probes were purchased from Integrated DNA Technologies (IDT, Coralvile, IA, USA). Conventional PCR reactions were 50 μL and were composed of 1 μL of each forward primer (Eub268), 1 μL of universal primer (1492R), 25 μL of EmeraldAmp Max PCR Master Mix (Takara, Japan), and 1 μL of genomic DNA from one of the three strains from the three phyla. Sterilized ultra-pure water was used to make up a total reaction volume of 50 μL. Genomic DNA from the three strains (*B. fragilis* GUT-04, *C. butyricum* TO-A, and *S. sonnei* KCCM 41282) was added separately. After confirming the specificity of each primer and probe set for their respective phyla, the sets were further evaluated by introducing a mock community (i.e., a mixture of the template DNA from the above-mentioned strains in equal proportions) into the MTq-PCR reaction. All PCR assays were performed with the following thermocycling program: 95 °C for 5 min; 35 cycles of 95 °C for 30 s, 60 °C for 30 s, and 72 °C for 30 s; and a final extension at 72 °C for 5 min. PCR mixtures using universal primers (both forward and reverse) served as positive controls. PCR mixtures without a template DNA served as negative controls.

### 4.7. Multiplex TaqMan qPCR

**Figure 6:**
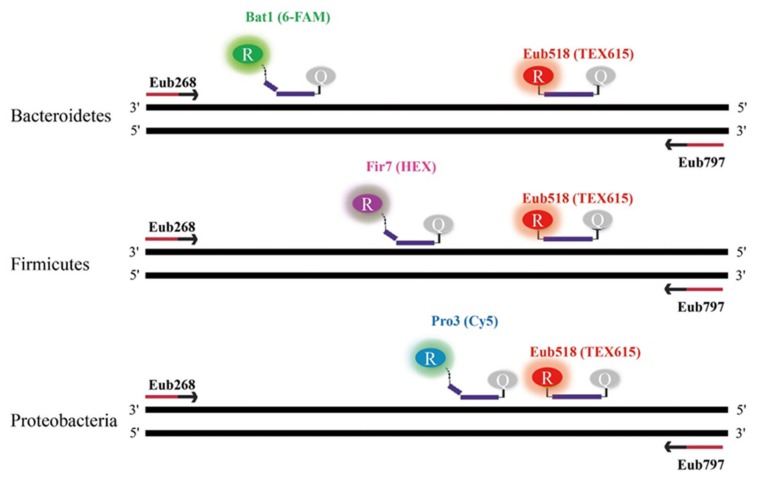
Schematic view of the multiplex TaqMan qPCR for simultaneous detection of the three dominant phyla in gut microflora (Bacteroidetes, Firmicutes, and Proteobacteria) in one PCR reaction.

In order to quantify the relative proportions of the three phyla (Bacteroidetes, Firmicutes, and Proteobacteria) in fecal samples from UC patients and healthy subjects, a multiplex, real-time TaqMan qPCR was carried out using a CFX96 (Bio-Rad, Hercules, USA). All qPCR reactions for both healthy and UC samples were performed in duplicate, and the average cycle threshold (Ct) values were calculated. Each qPCR reaction was 20 μL and was comprised of 10 μL of 2 X qPCR master mix (MGmed, Seoul, Korea), 1 μL of each primer, 0.5 μL of each probe, 1 μL of sample DNA, and sterilized ultra-pure water to make up a total reaction volume of 20 μL. Based on the conventional results, simultaneous quantification of the three phyla was performed with three TaqMan probes labeled with three distinct fluorophores. Bacteriodetes, Firmicutes, Proteobacteria, and Eubacteria probes were labeled with 6-FAM/BHQ1, HEX/BHQ1, Cy5/BHQ2, and TEX615/BHQ2, respectively (Figure 6). All qPCR reactions were performed using the following thermocycling program: initial denaturation at 95 °C for 5 min and 40 cycles of 30 s at 95 °C, 30 s at 60 °C, and 30 s at 72 °C. The relative proportions of Bacteroidetes, Firmicutes, and Proteobacteria were calculated as follows. The Ct values of each phylum were normalized by subtracting their respective Ct values from 40, and the resulting Ct values were summed to give a total Ct value. The relative abundance of each phylum was calculated by dividing their respective normalized Ct value with the total Ct value, and was expressed as a percentage by multiplying the resulting ratio by 100.

### 4.8. Statistical Analysis

A comparison of the relative abundances from NGS and TaqMan qPCR assays was conducted using Calypso version 8.84 [45]. The statistical similarity between NGS and TaqMan qPCR was performed using the analysis of similarities (ANOSIM) test. A dissimilarity analysis was performed using permutational manova (PERMANOVA, Adonis function) [46]. The similarity and dissimilarity analyses were computed based on Bray–Curtis distance.

## Figures and Tables

**Table 1:** Probe and primer design.

Oligonucleotide	Primer/Probe Name	Target Phylum	Fluoro-phore ^a^	Quencher	Sequence (5’–3’)	References
Probe	Bat1	Bacteroidetes	6-FAM	BHQ-1	GAGGCAGCAGTGAGGAATATTGGT	This study
	Fir7	Firmicutes	HEX	BHQ-1	AAGGCGACGATCGGTAGCCGRM ^*^	This study
	Pro3	Proteobacteria	Cy5	BHQ-2	GCCTTCGGGTTGTAAAGTACTTTCAGC	This study
	Eub518	Eubacteria	TEX615	BHQ-2	ATTACCGCGGCTGCTGG	[22]
Forward primer	Eub268	Eubacteria			TWGGYGRGGTAACGGCYCACCWA	This study
Reverse primer	Eub797	Eubacteria			GGACTACCAGGGTATCTAATCCTGTT	[23]

^a^ Fluorophores were coupled to the most effective probe candidates. ^*^ RM is an ambiguous nucleotide code where R stands for A or G, and M stands for A or C.

**Table 2:** In silico PCR assay with newly designed probes.

Probe	Target Taxon	Percent Coverage in the Target Taxon (%) ^a^		
		Perfect Match	1 Mismatch	2 Mismatches
**Bat1**	Bacteroidetes	63.7	85.2	93.0
	Firmicutes	0.0	0.1	3.7
	Proteobacteria	0.0	0.2	39.4
	Enterobacteriaceae	0.0	0.0	0.0
**Fir7**	Firmicutes	2.1	13.5	55.7
	Bacteroidetes	0.0	0.0	0.6
	Proteobacteria	0.0	0.1	3.9
	Enterobacteriaceae	0.0	0.0	0.1
**Pro3**	Proteobacteria	7.1	13.6	21.7
	Enterobacteriaceae	75.0	92.1	94.0
	Bacteroidetes	0.0	0.0	0.0
	Firmicutes	0.0	0.0	0.0

^a^ Percent sequence coverage in the target group based on the online “TestProbe” tool in the SILVA 138 SSU Ref NR database, released in January 2020.

**Table 3:** Comparison of our probe with previously reported Bacteroidetes-specific probes.

Probe	Sequence (5’–3’)	Location *	Gut Bacteroidetes (%) ^a^		All Bacteroidetes (%) ^a^	All Other Bacteria (%) ^a^	Reference
			*Bacteroides* spp.	*Provotella* spp.			
Bat1	GAGGCAGCAGTGAGGAATATTGGT	346–369	98.5	97.9	86.2	28.0	This study
S-P-Bdet-0107-a-S-21	GCACGGGTGMGTAACRCGTAT	107–127	91.9	95.9	78.7	23.7	[24]
CFB555f	CCGGAWTYATTGGGTTTAAAGGG	555–577	96.4	97.8	96.7	25.3	[25]
798cfbF	CRAACAGGATTAGATACCCT	779–798	97.8	98.5	98.2	25.3	[26]

* *Escherichia coli* position. ^a^ Percent sequence coverage in the target group based on the online “TestProbe” tool in the SILVA 138 SSU Ref NR database, released in January 2020.

**Table 4:** Comparison of probes from the current study with previously reported Firmicutes-specific probes.

Probe	Sequence (5’–3’)	Location *	Gut Firmicutes (%) ^a^		All Firmicutes (%) ^a^	All Other Bacteria (%) ^a^	Reference
			Clostridiaceae	Eubacteriaceae			
Fir7	AAGGCGACGATCGGTAGCCGRM	272–290	75.6	8.5	55.7	24.1	This study
S-P-Firm-0352-a-S-18	CAGCAGTAGGGAATCTTC	352–369	1.4	0.6	46.9	24.2	[24]
S-P-Firm-0525-a-A-18	ACCTACGTATTACCGCGG	525–542	98.2	95.5	96.6	80.1	[24]
Firm350f	GGCAGCAGTRGGGAATCTTC	350–369	95.7	96.0	87.3	72.2	[25]
928F-Firm	TGAAACTYAAAGGAATTGACG	905–925	94.2	93.8	96.2	96.4	[26]

* *Escherichia coli* position. ^a^ Percent sequence coverage in the target group based on the online “TestProbe” tool in the SILVA 138 SSU Ref NR database, released in January 2020.

**Table 5:** Comparison of probes from the current study with previously reported Proteobacteria-specific probes.

Probe	Sequence (5’–3’)	Location *	Gut Proteobacteria (Enterobacteriaceae) (%) ^a^	All Proteobacteria (%) ^a^	All other Bacteria (%) ^a^	Reference
Pro3	GCCTTCATACCACGGAGTACTTTCAGC	415–441	94.0	21.7	6.3	This study
Gamma395f	CMATGCCGCGTGTGTGAA	395–412	96.3	85.6	31.7	[25]
Gamma877F	GCTAACGCATTAAGTRYCCCG	859–880	0.3	9.3	31.8	[27]

* *Escherichia coli* position. ^a^ Percent sequence coverage in the target group based on the online “TestProbe” tool in the SILVA 138 SSU Ref NR database, released in January 2020.

**Table 6:** Comparison of the specificity between probes from the current study with those previously reported.

Target Phylum	Probe/Primer	Target Taxon	Percent Coverage in the Target Taxonomy (%)
Bacteroidetes	Bat1 *	Bacteroidetes	86.2
		Firmicutes	3.7
		Proteobacteria	39.4
		Enterobacteriaceae	0.0
	S-P-Bdet-0107-a-S-21	Bacteroidetes	78.7
		Firmicutes	13.9
		Proteobacteria	13.1
		Enterobacteriaceae	0.2
	CFB555f	Bacteroidetes	96.7
		Firmicutes	45.3
		Proteobacteria	0
		Enterobacteriaceae	0
		Bacteroidetes	99.4
	798cfbF	Firmicutes	99.0
		Proteobacteria	99.2
		Enterobacteriaceae	99.4
Firmicutes	Fir7*	Firmicutes	55.7
		Bacteroidetes	0.6
		Proteobacteria	3.9
		Enterobacteriaceae	0.1
	S-P-Firm-0525-a-A-18	Firmicutes	96.6
		Bacteroidetes	82.2
		Proteobacteria	80.2
		Enterobacteriaceae	93.2
	S-P-Firm-0525-a-S-18	Firmicutes	96.6
		Bacteroidetes	82.2
		Proteobacteria	80.2
		Enterobacteriaceae	93.2
	Firm350f	Firmicutes	87.3
		Bacteroidetes	14.3
		Proteobacteria	93.7
		Enterobacteriaceae	92.9
	928F-firm	Firmicutes	96.2
		Bacteroidetes	97.5
		Proteobacteria	95.6
		Enterobacteriaceae	92.8
Proteobacteria	Pro3*	Proteobacteria	21.7
		Enterobacteriaceae	94.0
		Bacteroidetes	0.0
		Firmicutes	0.0
	Gamma395f	Proteobacteria	85.6
		Enterobacteriaceae	96.3
		Bacteroidetes	2.3
		Firmicutes	21.0
	Gamma877F	Proteobacteria	9.3
		Enterobacteriaceae	0.3
		Bacteroidetes	1.2
		Firmicutes	33.4

* Probes designed in this study are indicated in bold.

**Table 7:** Adonis and Anosim statistical analyses between NGS and MTq-PCR using fecal samples collected from healthy subjects and UC patients.

Sample Source	Comparison	Adonis ^a^		Anosim ^a^	
		R^2^	*p* Value	R	*p* Value
Healthy	NGS vs. MTq-PCR	0.002	0.868	−0.049	0.779
UC	NGS vs. MTq-PCR	0.017	0.73	0.019	0.328
Across all samples	NGS vs. MTq-PCR	0.001	0.961	−0.04	0.89

^a^ Adonis and Anosim statistical analyses were carried out based on Bray–Curtis dissimilarities at the phylum level.

**Table 8:** Adonis and Anosim statistical analyses between NGS and MTq-PCR.

Method	Comparison	Adonis ^a^		Anosim ^a^	
		R^2^	*p* Value	R	*p* Value
MTq-PCR	Healthy vs. UC	0.745	<0.001	0.933	0.001
NGS	Healthy vs. UC	0.804	<0.001	0.984	0.001

^a^ Adonis and Anosim statistical analyses were carried out based on Bray–Curtis dissimilarities at the phylum level.

**Table 9:** Bacterial strains of different phyla used to design phylum-specific primer-probe sets.

Target Phylum	Strain	Accession Number
**Bacteroidetes**	*Bacteroides fragilis* ATCC 25285	KP326374
	*Bacteroides thetaiotaomicron* JCM 5827	NR_112944
	*Bacteroides coprophilus* CB42 (T)	AB260026
	*Prevotella copri* DSM 18205	NR_040877
	*Prevotella brevis* GA33	NR_041954
**Firmicutes**	*Ruminococcus gnavus* ATCC 29149	NR_036800
	*Clostridium difficle* ATCC 9689 (T)	NR_112172
	*Faecalibacterium prausnitzii* ATCC 27768 (T)	AJ413954
	*Enterococcus faecium* IMAU20967	MK369883
	*Roseburia hominis* A2-183 (T)	AJ270482
**Proteobacteria**	*Shigella sonnei* CECT 4887	FR870445
	*Enterobacter cloacae* ATCC 13047 (T)	NR_102794
	*Enterobacter aerogenes* ATCC 13048 (T)	KF516237
	*Escherichia coli* JCM 1649	AB242910
	*Pseudomonas aeruginosa* UTIRB3	MH910498

## Abbreviations

ANOSIM	Analysis of similarities
MTq-PCR	Multiplex TaqMan quantitative polymerase chain reaction
IBD	Inflammatory bowel disease
UC	ulcerative colitis
FMT	Fecal microbiota transplantation
NGS	Next generation sequencing
6-FAM	6-carboxylfluorescein
HEX	Hexachloro-fluorescein
Cy5	Cyanine dye
TEX615	Texas red fluorophore
BHQ-1	Black hole quencher-1
BHQ-2	Black hole quencher-1
